# Massachusetts Dental Society

**Published:** 1903-01

**Authors:** 


### MASSACHUSETTS DENTAL SOCIETY.

(Continued from Vol. XXIII., page 945.)

*President Faxon.*—The next thing in order on the programme is a paper by Dr. Walter F. Bisbee, of Camden, Me., entitled "Crown- and Bridge-Work to the Country Dentist," using W. H. Baird's method for making crowns, and other cheap methods.

(This address, having been given at the meeting of the Northeastern Association in October, 1901, is not incorporated in this report.)

*President Faxon.*—Dr. Bisbee has shown us how he carries on his work. Is there anything to be said in regard to the matters of interest in his paper? It certainly must contain many interesting points to some of you. Dr. Bisbee will be glad to answer any questions. Many of you heard Dr. Bisbee at the meeting of the Northeastern Association, I presume, and have benefited by his teaching.

*Dr. J. N. Crouse,* of Chicago.—The essayist is correct in stating that pulps usually die soon after the tooth is crowned. Some men attribute it to arsenic in the cement, but I am inclined to believe it is the phosphoric acid.

*Dr. J. T. Paul.*—I do not think that I quite got the idea of the cuttle-fish. Please explain it again.

*Dr. W. F. Bisbee.*—If I want to make a central or a lateral, I take a porcelain tooth up at that middle point. I press this into the cuttle-fish in this way. (Illustrating.) Then I put this ring over it. For a seamless crown you put it in endways.

*Dr. W. I. Brigham,* of Framingham.—I think that Dr. Bisbee prepares the crowns and has the cap properly fitted, and that is just the reason he has such good success; it is because he crowns them right and fits them properly; but the way many of the teeth are crowned, it is no wonder the pulps die. A great majority of the molars have the crowns laid on anywhere near the gum line. This is to me barbarous treatment.

*President Faxon.*—I think our experience will bear Dr. Brigham out. When the enamel is left on there is less danger of the pulp dying. Is there anything further, gentlemen?

*Dr. King.*—I would like to ask Dr. Crouse if treatment of the tooth with nitrate of silver where the enamel has been removed will prevent the action of the phosphoric acid on the pulp?

*Dr. J. N. Crouse.*—I think if carbolic acid or nitrate of silver were freely applied to the tooth before the band was put on there would be less danger of the pulp dying, but the most satisfactory and practical plan is to use an acid which will not irritate the pulp.

*Dr. Ned A. Stanley,* of New Bedford, Mass.—Chloroform acts as a preventive to some degree. It might destroy the action of the phosphoric acid.

*Dr. D. Hurlbut Allis,* of Springfield, Mass.—The better way yet is to use gutta-percha entirely, as we see a great many of our young men doing who are giving satisfactory crown- and bridge-work.

*Dr. Ned A. Stanley.*—While the question of the gold crown is under discussion, I wish to protest against its abuse; its easy adaptation has led to its too free use, and teeth that might be saved artistically have fallen victims to the gold cap, which at once is a monument to the inartistic skill of the dentist and a trade-mark for the patient to be known by among her friends. I refer more particularly to the capping of the incisor, canine, and bicuspid teeth for our lady patients.

Every dentist who is not familiar with the staple crown ought to become so as soon as possible. It is as durable for bridge-work, and avoids the unsightly glare of the gold cap, and you know it is the artistic as well as the practical which we are striving for.

*President Faxon.*—As the time is limited, I think I will ask Dr. Bisbee to close the discussion, and then a motion to adjourn will be in order. There is another paper at two o'clock. There will be time enough for us to get a light lunch, and I should recommend the same, as we are to have a banquet and I hope all will attend.

*Dr. Bisbee.*—I have nothing to say except that I believe in having very little gold show.

Adjourned.

*Afternoon Session.*

Session was opened by the President at 2.15 o'clock.

*President Faxon.*—The next paper to be presented before the Society is one not coming from the sublime to the ridiculous, but from the scientific to the practical. It is a subject about which there has been very little said in our meetings. It is "Management of Children in the Dental Office." It touches a little upon the psychological attitude of the dentist, and I am very much pleased to present to you Dr. Horace E. Eaton, of Toronto, Canada.

(For Dr. Eaton's paper, see page 8.)

DISCUSSION.

*President Faxon.*—To see Dr. Eaton stand up before us is enough to convince us that he understands the handling of children or any of his patients. To hear him read his paper would require but one word to impress upon us the fact that he is an expert in this line. He must have some of that imagination in children's stories that he has given children credit for. He has treated the subject in a psychological manner, and no doubt the susceptibilities of those who have heard him will receive their share of the good seed he has strewn around. The committee has been fortunate in procuring one to open the discussion on this subject who is a professor of psychology, who has taught throughout the country, as well as many dentists in Boston. I will ask Professor Barnes, Professor of Psychology, of the city of Boston, to favor us with some remarks.

*Professor William A. Barnes,* of Boston, Mass.—I feel much out of place here to-day, and not being accustomed to talk to an audience of this size, I have prepared a paper to read. At the end of this paper some of you may have questions you wish to ask, and I will take great pleasure in answering them.

The management of children in the dental office is a matter of keen knowledge of child-nature, and a fine application of tact as practically applied to dentistry. Dr. Eaton has thoroughly covered this subject in his able paper, and shown us how to apply general principles in the particular cases which he cited.

A practical working knowledge of just how to handle children in the dental office must be acquired and developed from actual experience. Not being a dentist, I can simply give you general principles in the art of control deduced from my own personal experience with men, women, and children in my particular profession.

A driver of high-bred trotting horses would scarcely expect to get valuable pointers in driving from a man who handles draught horses, ponies, or mules. You can readily understand that my relative position to you is that of the cart-driver to the expert jockey.

Were it not for the demonstrated fact that burley blacksmiths have become expert in mechanical dentistry, as well as remarkably successful in delicately handling men, women, and children in the dental office, we would not be here to-day discussing ways and means for mutual improvement; in a word, the Massachusetts Dental Society would not be in existence.

Since child-dentistry is an avowed necessity, it behooves every intelligent, progressive dentist to look about him for improvement in means, methods, and suggestions by which he may be enabled to perform the duty with credit to himself and his profession and with greater satisfaction and comfort to the little patient.

Now, the general principles underlying the scientific control of a being, be it horse, man, woman, or child, are identical. When one has thoroughly mastered a *working knowledge* of the general principles, all that remains to be done is to practically apply them to the particular work in hand.

It is now in order for me to give you a few general principles, which I will illustrate with incidents from my own daily experience, showing the practical application.

First. One is controlled by the strongest impression made upon the mind; in other words, one will do as he thinks, and not as others think.

Second. The mind can only be occupied fully with one idea or sensation at a time.

Third. The senses are the only avenues through which sensation can possibly reach the mind.

Fourth. The mind controls all emotional and physical activity.

Fifth. The mind of another is controlled principally by means of that subtle force best known as psychic power.

Sixth. Psychic power is expressed, or thrown out, by the operator's general appearance, gestures, facial expression, and verbal suggestions. Everything should combine and harmonize to convey the one leading idea which he wishes the patient to faithfully carry out.

Seventh. The physical expressions, verbal suggestions, and ideas of the operator must harmonize, or be adjusted to the one whose confidence he desires to obtain.

Success in influencing and controlling children, or, indeed, any one in any sphere of life, depends wholly upon the above-named general principles being put into intelligent practice.

One of my pupils, a physician, on coming to me for his second lesson, told me that he had been very successful in controlling his oldest boy psychologically, but had utterly failed to influence the younger one. He wished me to tell him why he had failed. I asked him to describe the nature of the two boys. When he had done so, I explained to him that the boy whom he had failed so entirely to influence was much more active in mind and body than the older one, and that he himself was too slow for the boy,—he bored him. In order to influence him, he must whip up, be more active in body and mind, and give the verbal suggestions more rapidly. He stated on his return, with much satisfaction, that he had been successful after following my suggestions in the case.

Another pupil, a lady, brought her youngest daughter upon whom to demonstrate and illustrate the lesson,—the control of one mind by another. I had successfully demonstrated the first lesson upon the mother, to her great satisfaction, and she had the utmost confidence in my ability, but not very much in her own. The mother and child of about ten years had scarcely seated themselves comfortably, when I tried to induce the child to talk about her school. As I had another pupil coming at the close of that hour, I naturally hurried the child too much. It being her first visit, and I an entire stranger to her, of course there was more rapid mentation than usual going on in her little brain. I fully realized these unfavorable conditions, but told her mother that I would try to influence her. I attempted to hold her spell-bound in her chair, but failed to do so, as I knew I must, considering existing conditions.

This failure surprised and excited the mother. She exclaimed, "Well, I do not see why you cannot influence my child; you influenced me."

I said to her, "Madam, your child is somewhat excited." "My child is not excited, and I want you to influence her," was the reply. "You do not understand me," I said. "Of course, your child is not excited in the broadest sense, but I am a stranger to her, the office is strange to her, consequently there is more or less rapid mentation going on in her brain. In other words, I have not taken the requisite amount of time to induce the most favorable state of consciousness for the reception of a new idea. If you will bring her the next time you come for a lesson, I will not be so hurried and will influence her for you." When the mother returned for her lesson she brought the child, as I had requested, and I was successful in controlling her to any extreme within a few minutes after she had entered the office.

These two attempts fixed indelibly a principle in the mind of the mother which I wished to impress upon her during the first lesson. In no other way could I have lodged this truth in her crude state of mind. The principle is to prepare the ground before attempting to sow the seed.

A gentleman engaged in the grocery business called upon me for general information. He told me that he did not have much faith in this power, yet, if there was anything in it by which he could increase his health and make him more successful in business, he might take it up. During our conversation he expressed great doubt as to there being anything in it for him. However, at the close of our talk he paid me one-half the amount for the full course of instruction, saying that he expected to leave the city for a week or two, and on his return would pay the remaining half and begin instruction. Two weeks, three weeks, four weeks, five weeks, six weeks rolled away, and still no sign of the gentleman. At the end of this time I wrote him suggesting that he begin his course of instruction before I left for my summer vacation. He came in answer to this, and said that he had not felt in good spirits, and had decided that he would not take up the instruction, that he preferred to lose what he had paid rather than risk losing a similar amount. I asked his reason for changing his mind. He said that a friend had discouraged him. I asked if his friend knew what he was talking about. He said he thought not, but was of the opinion that there was nothing in it. "Well," said I, "you take the advice of one who does know, pay the balance and proceed with the instruction immediately. You are the very man who sorely needs such instruction." The result of this encounter was that the gentleman was on hand promptly that same evening at seven o'clock for his appointment, but had again changed his mind. It took just about one minute to turn his thoughts in the direction that was surely for his own best interest. However, he still insisted that he did not believe in the influencing and controlling power. By way of answer, I called attention to the fact that his mind had gone through many changes, and that more than likely he would have to be knocked down and sat upon before he would realize the truth. Following this up, I influenced him so that he could not possibly open his eyes, and begged me to release them. Before he had time to forget the power of influence, I made him admit that he believed in it from personal experience, and that he was so thoroughly under my control that he was unable to resist it.

The most remarkable case which I will lastly and briefly mention, to impress upon you this matter of adjustment, is a patient whom I have at the present. I first learned of the case and was engaged to do something for him, if such a thing were possible, about the first of May. To use his mother's expression, "If you put him in a hole he would simply stay there." He was very morbid and ugly, and often wept for no apparent reason. No effort to get him out of doors or even near a window proved availing; nothing in the world had the slightest interest for him. My first visit was a brief one, about one minute, and my reception was not very cordial. I called every other day, gradually increasing the length of my stay, until at the present time I find it advisable to spend a half-hour with him each time. We take delightful walks together and have the most enjoyable talks on various subjects. Altogether the acquaintance has proved one of mutual benefit and pleasure. He has entertained me several times with sweet music, and his gentle manner is most attractive to me. He has been to see me at my office also.

Adjustment, harmony, naturalness, skilful suggestion, and love for mankind will explain it all.

Successful management of children depends as much or more upon what the operator does not say and do as upon what he does say and do.

1. Don't tell the child that you will not hurt him.
2. Don't talk too much.
3. Don't be excited or undecided in the presence of the child.
4. Don't let him see the instrument in your hand until absolutely necessary.
5. Don't make any false movements.
6. Don't ask the child if he wants his teeth attended to.
7. Don't make unnecessary remarks.

Following are a few things you might *tell him.*

1. Possibly your experience will be more pleasant than you think.
2. Come right along with me and we will make things just as comfortable as possible for you.

3. I think you are as agreeable and pleasant a boy as I ever saw.

If the dentist will eliminate those things which he should not do and say before children, his success in this particular line will be increased in the same proportion.

The extreme susceptibility of children often makes them more difficult to manage than adults, as the adjustment must be on a finer scale. This brings up the subject of self-control and perfect mastery of self. Study child nature, increase your psychic power, learn the law, and control your psychic force.

*President Faxon.*—Before closing, I would like to ask if there are any questions you would like to ask Professor Barnes. If not, I will ask Dr. Fillebrown to make a few remarks on this subject.

*Dr. Thomas Fillebrown,* Boston, Mass.—Mr. President, Dr. Eaton as a fairy man seems to beat us all. I was much interested in his very valuable paper. The question occurs to me, What are we fellows that cannot tell such nice fairy-tales going to do? Dr. Eaton has remarkable skill in this direction. He will conquer his patient every time, I do not doubt, yet I feel sure there are those of us who are not so ready, who have not got the fine qualification of the essayist. Now, what is this qualification of the operator? It is, I think, simply self-command, repose. Our essayist says never get mad, and if you do, keep it to yourself. We must not show ill-temper when we feel it. We can bring ourselves under such control that we will not have such a feeling towards any patient. A way to get this control is described in a small volume on "Power through Repose." It is one of the best books I have ever read. We all have experienced the difficulties the essayist has mentioned. We have simply got to be a little longer about the work. The boy who was nearly utterly incorrigible was soon brought under control by that quiet way of repose. According to my observation, instead of children's disliking to come to the dentist the first visit is a pleasure, but if you find a child who has had two or three unpleasant visits, you have a very different subject to deal with.

I do not think that it is necessary to keep the instrument out of sight. Let the children understand that they are the ordinary daily instruments of our work. I never thought of such a thing as keeping the instruments out of sight unless the child had been frightened beforehand. The best way is to look at the teeth, avoiding any disparaging remark, and just say to the child, "This will not hurt you to amount to anything. If it hurts a little, you will not care anything about it." Say to the child, "Just see how heavy you can make yourself. See how big you can breathe." You will be surprised how this way of proceeding will quiet yourself and the child too. I lately had a patient, a boy about six years old, and when I got him settled down I said to him, "Now, it will not hurt you," and in this case it did not at all. Now, here is a little plan that every one can use to-morrow. When you are about to commence to operate, just compose yourself by taking several long, deep breaths, ask your patient to do the same; you will be surprised to see how composing this simple thing is. It works just the same with adults as with children. Fear is the worst part. What you want is to allay those fears. What I have described is the way to do it. This is very simple, and you can all easily try it, and will succeed.

I at first believed in the necessity of shutting the patient's eyes so he could not open them without my permission. I soon found that I could make the suggestion effective without any special suggestion, until at the present time I very rarely hypnotize a patient. Possibly in such cases the patient is just as much hypnotized as if he were paralyzed in every muscle. Now, if this could be brought about, all we need for practical purposes is the personal, quiet influence of the operator on the patient. One case illustrates my point. I attended a meeting of the Vermont State Society some years ago while I was in my second stage, when I believed absolute hypnotism was not necessary. I asked for a patient for a clinic. A young boy was found whom I had not seen before, and who had little or no idea of what was wanted of him. I took an instrument and touched one of his teeth, and he nearly jumped out of the chair. I had to hold him down. I waited a moment or two, and then I placed my hand on his head and said to him, "This won't hurt you this time." Then I took an excavator and prodded all over the tooth and into some labial cavities, and he never winced. This is just a sample of my daily practice, and it can be the same with you. I hope you will try it. It is just as applicable to the child of one age as to another,—eight months, eight years, or eighty years.

Meeting adjourned till 6.30 P.M.

(To be continued.)

